# Evidence of SARS-CoV-2 Virus in the Middle Ear of Deceased COVID-19 Patients

**DOI:** 10.3390/diagnostics11091535

**Published:** 2021-08-25

**Authors:** Ionuț Isaia Jeican, Maria Aluaș, Mihaela Lazăr, Lucian Barbu-Tudoran, Dan Gheban, Patricia Inișca, Camelia Albu, Septimiu Tripon, Silviu Albu, Costel Siserman, Mihaela Laura Vica, Monica Muntean, Iulian Opincariu, Lia Monica Junie

**Affiliations:** 1 Department of Head and Neck Surgery and Otorhinolaryngology, University Clinical Hospital of Railway Company, Iuliu Hatieganu University of Medicine and Pharmacy, 400015 Cluj-Napoca, Romania; jeican.ionut@umfcluj.ro; 2 Department of Anatomy and Embryology, Iuliu Hatieganu University of Medicine and Pharmacy, 400006 Cluj-Napoca, Romania; iulian.opincariu@umfcluj.ro; 3 Department of Oral Health, Iuliu Hatieganu University of Medicine and Pharmacy, 400012 Cluj-Napoca, Romania; 4 Viral Respiratory Infections Laboratory, Cantacuzino National Military-Medical Institute for Research and Development, 050096 Bucharest, Romania; lazar.mihaela@cantacuzino.ro; 5 Electron Microscopy Laboratory, Faculty of Biology and Geology, Babeș-Bolyai University, 400006 Cluj-Napoca, Romania; lucian.barbu@ubbcluj.ro (L.B.-T.); tripon_septimiu@yahoo.com (S.T.); 6 Electron Microscopy Integrated Laboratory, National Institute for R&D of Isotopic and Molecular Technologies, 400293 Cluj-Napoca, Romania; 7 Department of Pathology, Iuliu Hatieganu University of Medicine and Pharmacy, 400015 Cluj-Napoca, Romania; dan.gheban@umfcluj.ro (D.G.); curta.camelia@umfcluj.ro (C.A.); 8 Department of Pathology, Emergency Clinical Hospital for Children, 400370 Cluj-Napoca, Romania; 9 Department of Pathology, County Emergency Hospital, 330084 Deva, Romania; patricia.bilei@gmail.com; 10 Imogen Medical Research Institute, County Clinical Emergency Hospital, 400014 Cluj-Napoca, Romania; 11 Department of Legal Medicine, Iuliu Hatieganu University of Medicine and Pharmacy, 400015 Cluj-Napoca, Romania; csiserman@umfcluj.ro; 12 Institute of Legal Medicine, Department of Legal Medicine, ‘Iuliu Haţieganu’ University of Medicine and Pharmacy, 400006 Cluj-Napoca, Romania; mvica@umfcluj.ro; 13 Department of Cell and Molecular Biology, Iuliu Hatieganu University of Medicine and Pharmacy, 400349 Cluj-Napoca, Romania; 14 Department of Infectious Disease, Clinical Hospital of Infectious Disease, Iuliu Hatieganu University of Medicine and Pharmacy, 400000 Cluj-Napoca, Romania; monica.muntean@umfcluj.ro; 15 Department of Microbiology, Iuliu Hatieganu University of Medicine and Pharmacy, 400349 Cluj-Napoca, Romania; mjunie@umfcluj.ro

**Keywords:** SARS-CoV-2, COVID-19, middle ear, otologic surgery

## Abstract

The presence of SARS-CoV-2 in the middle ear reveals the etiopathogenesis of otitis media in COVID-19, as well as an epidemiological risk during otologic examination and surgical procedures in COVID-19 patients. The study included 8 deceased patients with COVID-19. Tissue samples from the middle ear were subjected to virology, histopathology, scanning (SEM) and transmission (TEM) electron microscopy investigation. Ethmoidal mucosa samples were processed for virology analyses. qPCR resulted positive for 75% of nasal mucosa samples and 50% of middle ear samples. Ct values showed lower viral loads in middle ear samples. A proportion of 66.6% patients with positive results in the nasal mucosa showed positive results in the middle ear, and the subtype analysis of the complete genome sequences indicated B.1.1.7 lineage for all samples. In histopathological and SEM samples, no pathological aspects were identified. TEM revealed on the background of death critical alteration of cellular morphology, suggestive structures resembling SARS-CoV-2, goblet cells and immune cells. SARS-CoV-2 can be present in the middle ear of COVID-19 patients even if there is not clinical evidence of acute otitis media. Otolaryngologists could be particularly exposed to COVID-19 infection.

## 1. Introduction

Corona Virus Disease 19 (COVID-19) triggered a fight with uncertainty in the world scientific community. All around the world, the epidemiological situation of the COVID-19 pandemic is uncertain, and the Delta variant of Severe Acute Respiratory Syndrome-CoronaVirus-2 (SARS-CoV-2) is spreading to more and more countries [1]. 

Given the anatomical position of the upper respiratory tract mucosa at the interface with the surrounding air, it represents an important component of immunity. The direct anatomical connection of the middle ear to the nasopharynx through the eustachian tube permits transit of viruses and commensal nasopharyngeal bacteria to the middle ear and mastoid air cells [2,3]. 

At the beginning of the pandemic, a hypothesis was advanced that the middle ear and mastoid air cell system might be colonized with SARS-CoV-2 in positive patients, and it was recommended to delay non-urgent otologic surgery in these patients [4,5]. Fidan [6] reported the first case of otitis media in a COVID-19 patient in April 2020, and in October 2020, Frazier et al. [7] reported for the first time the isolation of SARS-CoV-2 from mastoid and middle ear specimens in deceased COVID-19 patients. In January 2021, Raad et al. [8] published a series of eight otitis media cases in COVID-19 patients, arguing that otitis media can be considered an associated manifestation of COVID-19. Thus, there is increasing evidence suggesting that otologic disorders, particularly hearing loss, may belong to the clinical spectrum of COVID-19 and, in some cases, may signal the presence of the disease [9].

Confirming the presence of SARS-CoV-2 in the middle ear is important both for elucidating the etiopathogenesis of otitis media in COVID-19 and for correctly evaluating epidemiological risk during otologic examination and surgical procedures in COVID-19 patients [10].

The aim of our study is to investigate the presence of SARS-CoV-2 virus in the middle ear of deceased COVID-19 patients and to perform its genetic characterization, as well as the histopathological and ultramicroscopic characterization of the middle ear mucosa in COVID-19. This is, to our knowledge, the second paper that supports the presence of SARS-CoV-2 by real-time polymerase chain reaction (RT-PCR) in necropsy samples from the middle ear, and the first article presenting the viral load in the nasal mucosa compared to the middle ear, viral genome analysis and microscopic findings. 

## 2. Materials and Methods

### 2.1. Study Design and Population

The samples were removed from deceased patients within the Department of Pathology of the County Emergency Hospital Deva, while complying with Romanian Law (Law 104/2003 on the handling of bodies and the removal of organs and tissues with a view to transplantation, and Government Decision No. 451/2004 on methodological norms for the application of law 104/2003) and specific international and national recommendations for COVID-19 [11,12,13,14,15]. The harvesting protocol for this study was approved by the County Emergency Hospital Deva Ethics Committee under No. 8942/04/02/2021 (nasal mucosa) and 17187/07/02/2021 (middle ear), and by the Administrative Staff of the County Emergency Hospital Deva under No. 8943/04/02/2021 (nasal mucosa) and 17188/07/02/2021 (middle ear).

Patients under 18 years of age; patients with nasogastric intubation, with known ENT pathologies (including allergic rhinitis), autoimmune diseases, cystic fibrosis or Kartagener syndrome; pregnant women and patients with insufficient samples were excluded from the study. In addition, medical data about clinical information, demographics and results of laboratory tests were collected from their medical records file. 

### 2.2. Sampling

Five tissue samples from the middle ear were obtained 12 h after death, by transtympanic curettage with a Volkmann curette No. 2, using appropriate protective measures: one for SARS-CoV-2 virology analyses (stored in a viral transport medium BioSci virus sampling tube model FBY, Darkewe Biotech Co. Ltd., Shenzhen, China), where the tubes were stored immediately after collection in a freezer at a temperature of −20 °C, and then they were transferred to a freezer at a temperature of −80 °C over the next 24 h and stored until analysis); one for histopathology (stored in formaldehyde 7%); and two for electron microscopy – scanning and transmission, respectively (stored in glutaraldehyde 2.7%). The samples for electron microscopy were sent for processing immediately after collection. 

The tissue samples from the nasal mucosa were obtained by ethmoidal curettage with a Volkmann curette No. 2, using appropriate protective measures, for virology analyses.

### 2.3. Virology Analysis for SARS-CoV-2

#### 2.3.1. Antemortem PCR

Combined throat/nasal antemortem sampling (with a collection device produced by Sanimed International Implex, Bucharest, Romania) was performed for RT-PCR SARS-CoV-2 (QuantStudio v5 analysis method with TaqPath COVID-19 CE-IVD RT-PCR Kit, The QuantStudioTM Design & Analysis Software, Thermo Fisher Scientific, Pleasanton, CA, USA) in the laboratory of County Emergency Hospital.

#### 2.3.2. Tissue Virology Analyses

Total RNA isolation was performed with NucleoSpin RNA for tissue (Macherey-Nagel, Dueren, Germany) and MagnaPure 96 DNA and Viral NA from viral transport medium, according to the manufacturer’s instructions. The Allplex 2019-nCoV assay (Seegene, Seoul, Korea) was designed for amplifying three viral targets: E gene (specific to the subgenus Sarbecovirus), N and RdRP genes (both specific to SARS-CoV-2), using a QuantStudio 7 Pro Real-Time PCR System (Applied Biosystems, Waltham, Massachusetts, USA) and/or a Bio-Rad CFX96 instrument (Bio-Rad, Hercules, CA, USA). All SARS-CoV-2 RNA-positive samples (both from the ethmoidal mucosa and from the middle ear) underwent real-time whole-genome sequencing. RNA preparation and amplification were done in accordance with protocols published by the ARTIC network using the V3 version of the ARTIC primer set from Integrated DNA Technologies (Coralville, IA, USA) to create tiled amplicons across the SARS-CoV-2 genome. Libraries were prepared using the Nextera DNA Flex library preparation kit and MiSeq reagent cartridge V2 (Illumina, San Diego, CA, USA) [16]. All results were analyzed by the same investigator (M.L.).

### 2.4. Histopathology

The samples were fixed in formaldehyde 7% for 5 days, after which the samples were oriented and placed in cassettes. Tissue processing was performed using a vacuum infiltration processor, Tissue-Tek VIP 5 Jr (Sakura, Alphen aan den Rijn, The Netherlands). Paraffin embedding and sectioning were performed using the Tissue-Tek TEC 6 system (Sakura, Alphen aan den Rijn, The Netherlands) and Accu-Cut SRM 200 Rotary Microtome (Sakura, Alphen aan den Rijn, The Netherlands). Slide staining was performed using the automated slide stainer Tissue-Tek Prisma Plus (Sakura, Alphen aan den Rijn, The Netherlands), according to the internal staining protocol, using Mayer Modified Hematoxylin (Titolchimica, Rovigo, Italy) and Eosine solution (10 g Eosine B in 1000 mL distilled water). For Gram staining, the Gram Stain Kit (Gram Fuchsin Counterstain) (Atom Scientific, Manchester, UK) was used.

Microscopic examination was performed by the same experienced pathologist (D.G.), using an Olympus BX46 clinical microscope (Olympus Europe SE & Co., Hamburg, Germany) with the dedicated image acquisition camera and software. 

### 2.5. Scanning Electron Microscopy (SEM)

The samples were fixed in glutaraldehyde 2.7% for 2 h, washed with phosphate buffered saline (PBS) and then with distilled water, and were then left to dry. The dried samples were glued to a support with silver paste and sputter coated with a 10 nm thick Au layer before imaging (Agar Auto Sputter Coater, Agar Scientific Ltd., Stansted, Essex, UK). Scanning electron microscopy (SEM) was conducted on a Hitachi SU8230 cold field emission gun (Tokyo, Japan) at 30 kV. All samples were examined by the same experienced investigator (L.B.T.).

### 2.6. Transmission Electron Microscopy (TEM)

The tissues fixed in glutaraldehyde (2.7% in 0.1 M PBS) for 120 min were rinsed 3 times with 0.15 M PBS, for 1 h each, and postfixed in 2% osmium tetroxide. Dehydration was accomplished in a series of mixtures (acetone 30, 50, 70, 80, 90, three times 100%). Inclusion was made in Epon 812 (EMS USA, Electron microscopy Sciences). The dehydrated tissue was then placed in a polymerization mixture according to the manufacturer’s protocol and left overnight at room temperature for final mixing and embedding. Polymerization was performed in a freshly prepared mixture of the above composition for 2 days at 55 °C. Ultrathin sections, about 90 nm thick, were obtained using a Leica- UC7 ultramicrotome and a diamond knife (Diateome, Swiss) (Leica Microsystems, Bensheim, Austria). The sections were collected on copper grids covered by a thin layer of Formvar. Final staining of the sections included treatment with Uranyless (Agar Scinetific, UK) for 2 min and with lead citrate for 2 min. Transmission electron microscopy (TEM) was conducted on a Jeol 1010 cold field emission gun (Tokyo, Japan). All samples were examined by the same experienced investigator (L.B.T.).

## 3. Results

The patients included in the study group were aged between 56–87 years (mean age 73 years) (Table 1), with 62.5% (*n* = 5/8) men and 37.5% women (*n* = 3/8). No patient complained of otologic symptoms during admission, according to their medical records.

### 3.1. Virology Analyses

A total of 16 samples (8 from the nasal mucosa and 8 from the middle ear mucosa) were tested using qPCR assay. In the nasal mucosa, 75% (*n* = 6/8) of samples were positive, while in the middle ear, 50% (*n* = 4/8) were positive. Thus, 66.6% (*n* = 4/6) of cases with a positive result in the nasal mucosa also had a positive result in the middle ear. 

Ct values ranged from 17 to 37, with higher Ct values (lower viral loads) in the ear samples (Table 1). 

Complete genome sequences were amplified both from the nasal mucosa sample and from the middle ear sample for 4 patients (50%, *n* = 4/8), and for one case, complete genome sequences were amplified only from the nasal mucosa sample (case No. 3, Table 1).

The subtype analysis indicated that all sequences belonged to lineage B.1.1.7, defined by multiple amino acid changes including three deletions (69–70 del and 145 del) and seven mutations (N501Y, A570D, D614G, P681H, T716I, S982A and D1118H) in the spike protein. All five sequences were uploaded in the GISAID database under accession numbers: EPI_ISL_2727499, EPI_ISL_2727500, EPI_ISL_2727566, EPI_ISL_2727501 and EPI_ISL_2652169.

### 3.2. Histopathology

In all collected samples, the middle ear mucosa with a thin, flat, non-secretory squamous epithelium, and areas of ciliated columnar cells were observed. In the subepithelial layer, loose connective tissue was seen. In addition, bone fragments, rests of hairs, cerumen and keratin debris, due to the harvesting procedure, could be identified. No pathological element was detected in any of the samples: presence of inflammatory cells, edema, viral cytopathic effect, basal cell hyperplasia, metaplasia or other epithelial changes.

### 3.3. SEM

**Figure 1:**
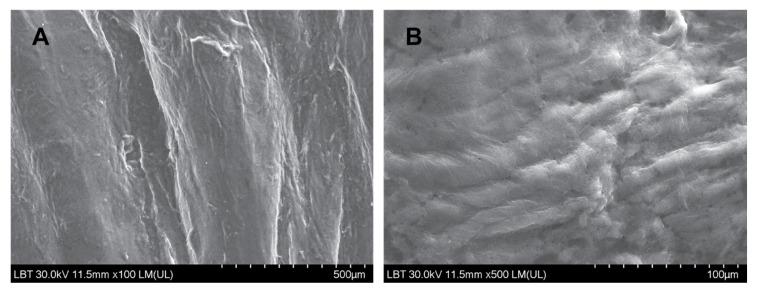
(**A**,**B**)—Ultramicroscopic aspects of the middle ear mucosa in deceased COVID-19 patients (SEM): flat epithelial surface, without microbial presence or surface immune cells.

In all collected samples, the flat epithelial surface without microbial presence or surface immune cells in any of the samples was observed (Figure 1) (the folded appearance of the mucosa is due to the curettage procedure).

### 3.4. TEM

**Figure 2:**
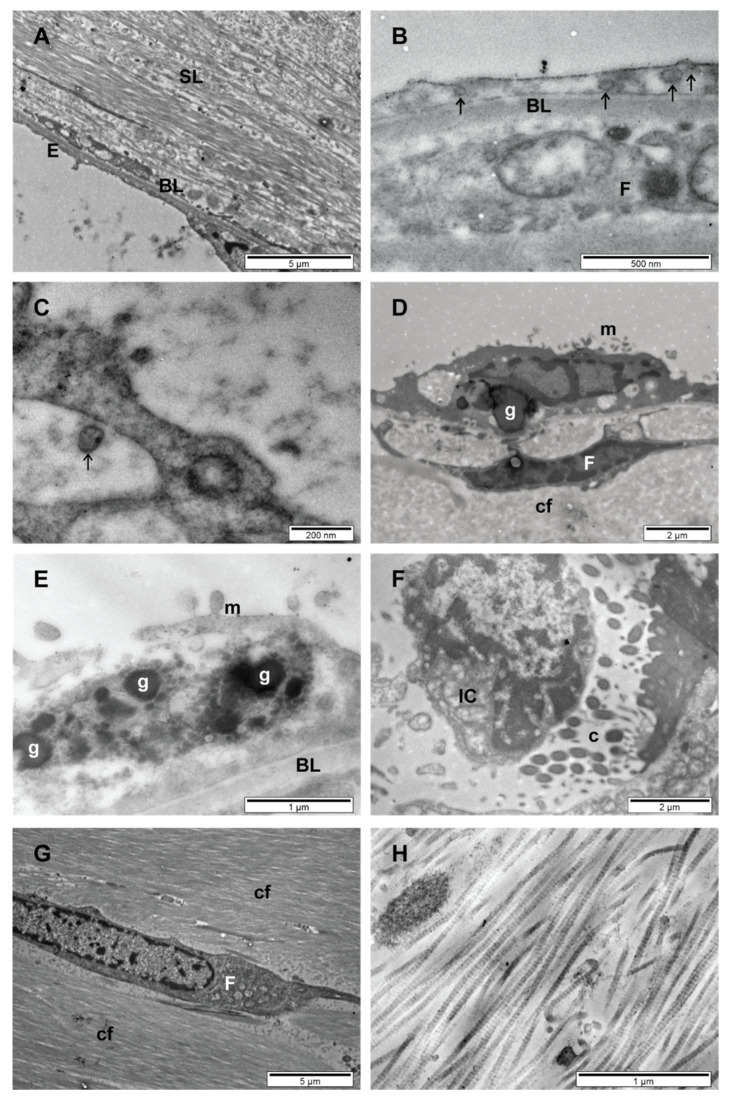

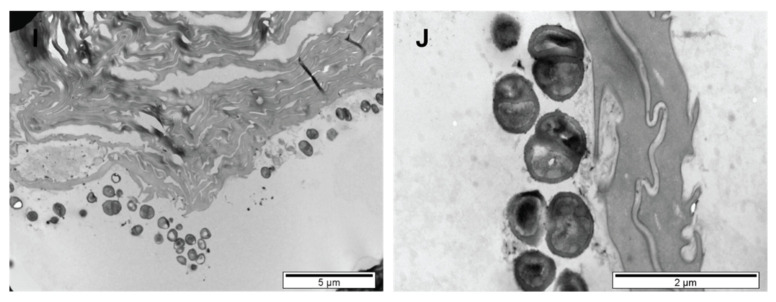
Ultramicroscopic aspects of the middle ear mucosa in deceased COVID-19 patients (TEM): (**A**)—Epithelium and subepithelial layer; (**B**,**C**)—Structures suggestive of SARS-CoV-2 virus in epithelial cells (death critical alteration of cellular morphology) (arrows); (**D**,**E**)—Goblet cells with secretory granules; (**F**)—Transition area from simple squamous epithelium to respiratory epithelium, guarded by an immune cell; (**G**)—Fibrocyte surrounded by collagen fibers in the subepithelial layer; (**H**)—Collagen fibers in the subepithelial layer; (**I**,**J**)—Bacterial aggregates on debris (external surface of the tympanic membrane); (E—Epithelium, BL—Basal lamina, SL—Subepithelial layer, F—Fibrocyte, cf—Collagen fibers, g—Secretory granules, m—Microvilli, c—Cilia, IC—Immune cell).

The ultramicroscopic TEM aspects of the middle ear mucosa in deceased COVID-19 patients are presented in Figure 2.

Given the 12 h elapsed from death to sampling, almost all samples showed death critical alteration of cellular morphology, cell lysis and autophagic vacuoles. Under these circumstances, the identification of SARS-CoV-2 virus cannot be affirmed, but we show the presence of suggestive structures resembling SARS-CoV-2 (enveloped particles with double contour membrane and with projections on the surface, and a heterogeneous, electron-dense, granular interior) (Figure 2B,C). 

In the epithelium, goblet cells (Figure 2D,E) and immune cells (Figure 2F) were detected. In the subepithelial layer, we identified fibrocytes surrounded by collagen fibers (Figure 2G,H). On the external surface of the tympanic membrane, we observed bacteria (Figure 2I,J), which were most probably commensals of the external auditory canal.

## 4. Discussion

COVID-19 is a respiratory infection with early nasal pathogenesis, which can extend to systemic damage. Nasal cells are the site of the first step of infection; these cells express the highest levels of angiotensin-converting enzyme 2 (ACE2) and of the cellular serine protease TMPRSS2, the main entry receptors for SARS-CoV-2 [3,17]. ACE2 and TMPRSS2 are also present in the eustachian tube, middle ear and cochlea, suggesting that these tissues are susceptible to SARS-CoV-2 infection [18]. Furthermore, the congestion of the turbinates and eustachian tube can promote the transition of viruses and bacteria in the middle ear [19].

The presence of nucleic acids of respiratory viruses, including other human coronaviruses, has been identified in middle ear effusions by PCR [20,21]. Frazier et al. [7] reported for the first time the isolation of SARS-CoV2 from both mastoid and middle ear samples in 33.3% (*n* = 1/3) cases of deceased COVID-19 patients, and in another case, the virus was isolated only from the right middle ear. We isolated the virus from the middle ear mucosa in 50% of cases (*n* = 4/8). We chose curettage as a sampling method because it involves a lower contamination risk compared to the otologic approach by craniotomy [22].

The cycle thresholds for middle ear samples are similar between the two studies, indicating a moderate to high viral load; Frazier et al. [7] reported 24 to 36, and in our study, we obtained 22 to 37. For all our positive samples, analyses of genome sequences revealed Lineage B.1.1.7 (20I/501Y.V1), Alpha. This variant has been circulating in the United Kingdom since at least 20 September 2020 (VOC 202012/01). There is evidence of a higher transmissibility compared to other lineages, resulting in rapid growth in the United Kingdom and internationally despite measures being in place [23]. 

The mastoid air cells directly communicate with the middle ear through the aditus ad antrum, so that mastoid viral load can be like that of the middle ear mucosa [21]. However, the results published by Frazier et al. [7] show a lower viral load in the mastoid than in the middle ear, while our results suggest a lower viral load in the middle ear compared to the nose.

Mucosal immunological defense in the middle ear and the Eustachian tube utilizes both physicochemical barriers (mucus and mucosal epithelial cells) and innate immune responses (inflammation, cellular infiltration, effusion and antimicrobial protein secretions), in addition to adaptive host immune responses [2]. 

Certainly, viral infection of the upper airway mucosa results in interruption or dysregulation of normal inflammatory responses of the mucosa within the eustachian tube and middle ear. The middle ear mucosa frequently responds immunologically during upper airway infections by innate inflammatory reactions [2]. The viral infection of the upper respiratory mucosa may initiate an inflammatory cascade in the middle ear mucosa, with the secondary development of acute otitis media [24]. Adenovirus, respiratory syncytial virus, influenza and parainfluenza virus, coronavirus, enterovirus and rhinoviruses are commonly associated with otitis media [25]. However, the pathogenesis of acute otitis media involves a complex interaction between respiratory viruses, bacteria and host inflammatory response [26,27]. 

In addition to eliciting local and systemic immune-inflammatory responses, viral infection of the upper respiratory mucosa appears to have a substantial impact on the nasopharyngeal bacterial flora [26]. Despite the multitude of studies regarding the interaction between viruses and bacteria, the detailed mechanisms by which the bacteria that colonize the nasopharyngeal mucosa become pathogenic and invasive during viral respiratory infection are still unknown. It seems that through different mechanisms, viral infection activates epithelial cells; subsequently, bacterial adherence to epithelial cells increases [26,28]. Clinical and experimental studies have shown that pneumococcal colonization of the nasopharynx increased after infection with influenza A virus [29,30]. Previous studies by our team showed that the nasal mucosa of deceased COVID-19 patients presents numerous microbial aggregates (especially bacteria and/or fungi), suggesting nasal dysbiosis (data in the process of being published).

In addition, viral infection can increase the rate of mucus secretion and can alter fluid viscosity through upregulation of gel-like mucin secretion [31]. It has been demonstrated that influenza A virus can reduce the number and activity of epithelial cell cilia within the eustachian tube, and secondarily, it reduces the capacity for neutralizing or clearing otopathogens from the middle ear [32].

In practice, histological exam is not performed for middle ear inflammation outside of research studies. The epithelium of the middle ear mucosa is thin, flat, non-keratinized, squamous and non-secretory (Figure 2A), and it is disrupted by ciliated columnar cell islets (Figure 2F), which form the mastoid-tubo-tympanic mucociliary clearance pathway. Goblet cells (Figure 2D,E) are scattered among the squamous cells near the eustachian tube orifice [33,34].

The subepithelial layer consists of fibrocytes (Figure 2D,G), collagen fibers (Figure 2H), blood and lymph capillaries and nerve fibers [35,36,37]. Microscopic studies have evidenced few immune cells in the healthy middle ear mucosa [2]. In only one COVID-19 sample, we identified an immune cell (Figure 2F) that guarded the transition area from the simple squamous epithelium to the respiratory epithelium, possibly in the vicinity of the eustachian tube. The tympanic membrane presents a thin fibrous structure lined by a single flat layer of cuboidal epithelium on the middle ear side [38].

Histopathological aspects vary depending on the severity of the disease; edema and hyperemia of the subepithelial space are followed by infiltration of polymorphonuclear leukocytes (purulent stage). Subsequently, mucosal metaplasia develops (the epithelium changes from flat cuboidal to pseudostratified, columnar or cuboidal, with proliferation of goblet cells), and granulation tissue forms [39,40]. We identified no histopathological aspects in our samples.

Coronaviruses can be confused in TEM samples with normal cell organelles, and autolysis of cells can complicate morphological assessment [41]. SARS-CoV-2 viruses are enveloped particles with a double contour membrane, with projections on the surface, and a heterogeneous, electron-dense, partly granular interior; intracellular particles are typically located within membrane compartments [42,43,44]. We identified particles suggestive of SARS-CoV-2 (Figure 2B,C), without being able to identify it with certainty. 

Otitis media can be either a clinical manifestation or a complication of COVID-19. Clinical otologic disorders, especially hearing loss, may signal the presence of COVID-19 disease [8,45].

Additionally, mastoid and middle ear colonization with SARS-CoV-2 does not necessarily imply otologic symptomatology [7], as our results also show. Mohan et al. [46] reported a 23-year-old male with complicated acute otitis media with facial paralysis, acute infection with SARS-CoV-2 and a negative PCR of the middle ear fluid.

The certain proof of the presence of SARS-CoV-2 virus in the middle ear has implications for otologic surgeons and staff who handle surgical equipment, instruments, suction tubing and canisters [7]. Surgery of the middle ear and mastoid (especially mastoidectomy performed using a high-speed drill) is considered an aerosol generating procedure and presents a similar risk to other similar procedures: endotracheal intubation, tracheostomy and surgery of the airway [47,48]. If the virus is present in aerosols, the risk of transmission of SARS-CoV-2 to the operating room staff is significant. Blood particles and bone dust circulate in the form of aerosols during high-speed drilling, and the corneal route of transmission is possible. Loupes and the operative microscope do not provide sufficient protection [49].

Using cold surgical instruments, increasing the use of curettes and microdebrider use pose a significantly lower aerosolization risk than a high-speed drill [50]. Transcanal endoscopic ear surgery may avoid mastoidectomy. Unlike an operative microscope, transcanal endoscopic ear surgery allows the use of eye protection (goggles, face shields) [51].

Taking into consideration the high rate of asymptomatic patients [52], non-urgent cases should be delayed until preoperative testing, and non-tested urgent cases should be considered as suspect positive with a risk of exposure to SARS-CoV-2. Enhanced personal protective equipment will be used and powered instrumentation will be employed only when necessary, while air-purifying respirator or sealed eye protection is recommended [4,53]. In an otologic surgical intervention, there only needs to be a single surgeon in the room and all visitors should be removed; similarly, in the ear clinic, visits should be restricted [5].

The current study had several limitations. In the first place, the number of cases was relatively small. Secondly, the lack of sampling from the mastoid was another limitation. Thirdly, the absence of Ct values from combined throat/nasal RT-PCR for SARS-CoV-2 did not allow us to correlate these values with those obtained in nasal and middle ear samples.

## 5. Conclusions

In COVID-19 patients, the SARS-CoV-2 virus may be present in the middle ear even when these patients do not develop acute otitis media, a situation in which viral load is lower than that of the nasal mucosa and there are no histopathological changes in the middle ear mucosa.

Otolaryngologists are particularly exposed to COVID-19 infection from the nose or middle ear. The certain presence of SARS-CoV-2 in the middle ear has significant implications for otolaryngology procedures.

Further experimental research is required to study the interaction mechanisms between SARS-CoV-2 and local defense mechanisms, on the one hand, and between SARS-CoV-2 and the local microbiota, on the other hand.

## Figures and Tables

**Table 1:** Results of virology analyses.

No. of Samples, Sex, Age	Ct Values Nasal Mucosa (E/RdRp/N)	Ct Values Middle Ear (E/RdRp/N)	Genome Sequences	GISAID
1, M, 56	-/-/37	-/-/-	-	-
2, M, 75	34/37/34	-/-/-	-	-
3, M, 87	24/25/22	-/-/37	Lineage B.1.1.7 (20I/501Y.V1), Alpha	EPI_ISL_2727499
4, F, 68	26/23/26	32/25/31	Lineage B.1.1.7 (20I/501Y.V1), Alpha	EPI_ISL_2727500
5, M, 80	20/17/20	24/23/24	Lineage B.1.1.7 (20I/501Y.V1), Alpha	EPI_ISL_2727566
6, M, 58	-/29/33	29/25/28	Lineage B.1.1.7 (20I/501Y.V1), Alpha	EPI_ISL_2727501
7, F, 82	-/-/35	-/-/35	-	-
8, F, 78	21/19/23	27/22/26	Lineage B.1.1.7 (20I/501Y.V1), Alpha	EPI_ISL_2652169

## Data Availability

The virology analysis results are available at the Viral Respiratory Infections Laboratory, Cantacuzino National Military-Medical Institute for Research and Development, 050096 Bucharest, Romania; Contact: lazar.mihaela@cantacuzino.ro. The results of Histopathologic and Electron Microscopy Exams are available at the Department of Anatomy and Embryology, Iuliu Hatieganu University of Medicine and Pharmacy, 400006 Cluj-Napoca, Romania; Contact: jeican.ionut@umfcluj.ro.
